# *Parkia speciosa* Hassk.: A Potential Phytomedicine

**DOI:** 10.1155/2013/709028

**Published:** 2013-07-17

**Authors:** Yusof Kamisah, Faizah Othman, Hj Mohd Saad Qodriyah, Kamsiah Jaarin

**Affiliations:** ^1^Department of Pharmacology, Faculty of Medicine, UKMMC, Universiti Kebangsaan Malaysia, Jalan Raja Muda Abdul Aziz, 50300 Kuala Lumpur, Malaysia; ^2^Department of Anatomy, Faculty of Medicine, UKMMC, Universiti Kebangsaan Malaysia, Jalan Raja Muda Abdul Aziz, 50300 Kuala Lumpur, Malaysia

## Abstract

*Parkia speciosa* Hassk., or stink bean, is a plant indigenous to Southeast Asia. It is consumed either raw or cooked. It has been used in folk medicine to treat diabetes, hypertension, and kidney problems. It contains minerals and vitamins. It displays many beneficial properties. Its extracts from the empty pods and seeds have a high content of total polyphenol, phytosterol, and flavonoids. It demonstrates a good antioxidant activity. Its hypoglycemic effect is reported to be attributable to the presence of **β**-sitosterol, stigmasterol, and stigmast-4-en-3-one. The cyclic polysulfide compounds exhibit antibacterial activity, while thiazolidine-4-carboxylic acid possesses anticancer property. The pharmacological properties of the plant extract are described in this review. With ongoing research conducted on the plant extracts, *Parkia speciosa* has a potential to be developed as a phytomedicine.

## 1. Introduction

**Figure 1:**
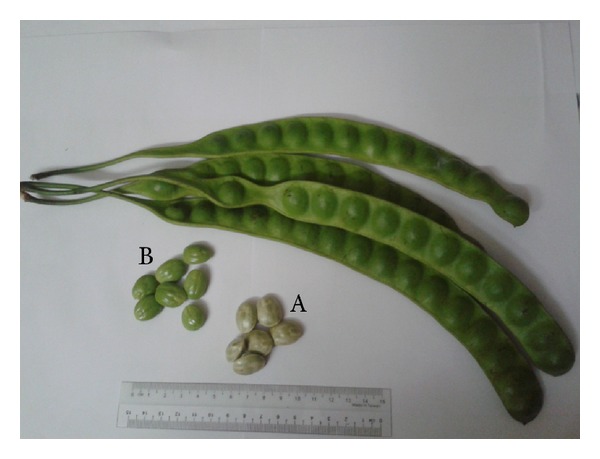
The pods and seeds with (grey, A) or without (green, B) seed coats of *Parkia speciosa*. The plant materials were collected from a plantation at Batang Kali, Selangor, Malaysia, in January 2013.

*Parkia speciosa* Hassk., or stink bean, is a plant that is abundantly found in the tropical regions like Malaysia, Indonesia, Thailand, and Philippines [1, 2]. It is a plant that belongs to the genus *Parkia* and species *speciosa* in the family Fabaceae (also placed in Leguminosae and Mimosaceae). It is known as petai in Malaysia, Singapore, and Indonesia [1, 3], sator or sataw in Thailand [4, 5], u'pang in Philippines [3], and yongchak in India [6]. It grows up to 40 meter high [6]. It bears green long and flat beans which are called pods in stalks. The stalks are 2 to 6 cm wide and 30 to 45 cm long. The light green stink bean seeds with seed coats are encapsulated in these pods (Figure 1). The seeds have a peculiar smell and can be eaten raw as “ulam” (a Malay word for uncooked) or cooked. The seeds are the most consumed “ulam” in Malaysia [7]. Half-riped seeds are also usually pickled in brine. The plant seeds have been used by the locals to treat various diseases and symptoms like diabetes, kidney disorder, and headache [1, 8, 9].

## 2. Nutritional Values

*P. speciosa* seeds contain many nutritional values such as protein, fat, and carbohydrate. They are also a good source for minerals (Table 1). The seeds have a considerable amount of vitamin C [10] and *α*-tocopherol (vitamin E) [11]. Among fourteen types of vegetable which were commonly consumed by the southern Thais, *P. speciosa* seeds had relatively the highest content of thiamin (vitamin B_1_, 2.8 *μ*g/g) but insignificant antithiamine factor [12]. High concentration of tannin was detected in its seed coats and pods compared to other fruit vegetables [5]. Tannin has been reported to decrease protein and amino acid digestibility [13]. Therefore, it is not advisable for children to consume the seeds in high amounts as good protein absorption is necessary for good body development.

## 3. Chemical Compounds

Table 2 tabulates the phytochemicals screening in various parts of *P. speciosa*. Almost all major chemical compounds are present in the seeds. Phenolic compounds are also present in almost all parts of the plant. To date, not many studies have been done to elucidate the chemical properties in the pods. In the seeds, the terpenoids detected using gas chromatography were *β*-sitosterol, stigmasterol, lupeol, campesterol, and squalene [14, 15]. Interestingly, lupeol was found to possess anticarcinogenic [16], antinociceptive, and anti-inflammatory properties [17]. No flavonoid like quercetin, myricetin, luteolin, kaempferol, or apigenin was detected in the methanolic extract of *P. speciosa* seeds using a reversed-phase high performance liquid chromatography [18], but it was noted to be present in the ethanolic extract when screened using a colorimetric assay [10]. Besides, alkaloids and saponins were also found in the plant. The seeds also contain cyclic polysulfides, namely, hexathionine, tetrathiane, trithiolane, pentathiopane, pentathiocane, and tetrathiepane [19] which are responsible for its strong pungent smell and taste, while the presence of djenkolic acid in the seeds is thought to cause blockage of the ureter [20]. 

**Figure 2:**
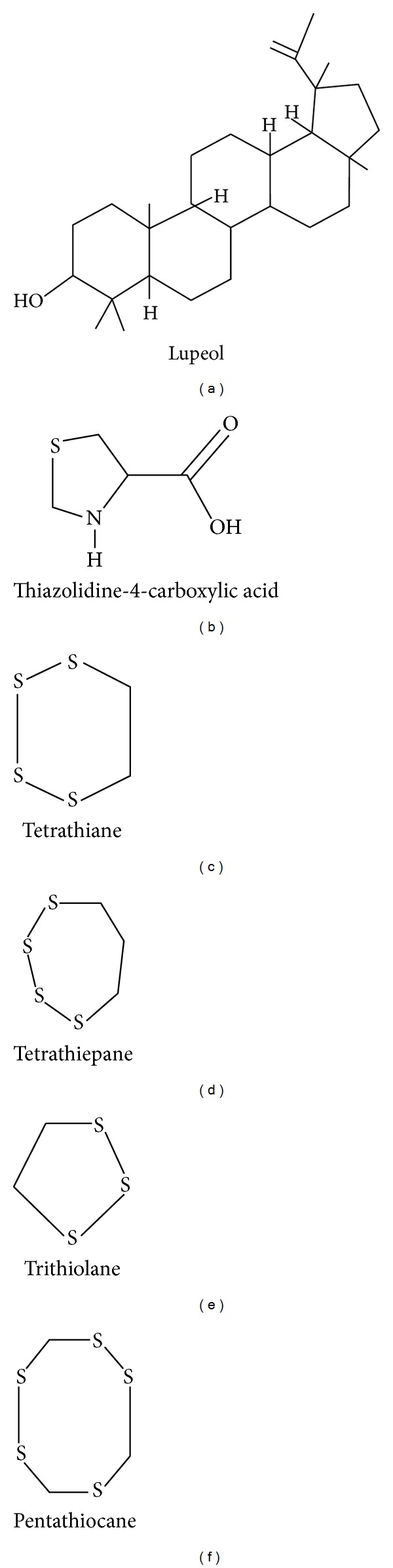
Chemical structures of lupeol (a), thiazolidine-4-carboxylic acid (b), and some cyclic polysulfides (c-f) found in *Parkia speciosa*.

Chromatography analysis of the stink bean seeds had identified presence of fatty acids which were undecanoic, myristic, palmitic, oleic, linoleic, elaidic, stearic, stearoic, lauric, arachidonic, and linoleic acids [14]. In the seeds, formation of thiazolidine-4-carboxylic acid, a thioproline, was remarkably increased after boiling when detected using gas chromatography-thermal energy analyzer [21]. This compound was reported to have an anticarcinogenic property [22]. The chemical structures of lupeol, thiazolidine-4-carboxylic acid, and some cyclic polysulfides (tetrathiane, tetrathiepane, trithiolane, and pentathiocane) are shown in Figure 2.

## 4. Properties of *P. speciosa*

Studies conducted on *P. speciosa* have revealed many potential properties of the plant. However, it needs to be ventured further to understand and to identify its mechanism of actions for future use in humans. Each property of the plants is described and discussed. 

### 4.1. Antioxidant Activity

Oxidative stress has been implicated to play an important role in many pathological conditions such as hypertension [23], hyperbilirubinemia [24], stress-induced gastric lesion [25], hyperhomocysteinemia [26], cancer [27], atherosclerosis [28], and diabetes [29]. Therefore, there is a growing interest to study plants with potential antioxidant property to treat various diseases. *P. speciosa* is not an exception.

Commonly, the simple ways to measure natural antioxidant in plant extracts are the total phenolic content, reducing ferric ion antioxidant potential (FRAP) and 1,1-diphenyl-2-picrylhydrazyl (DPPH) free radical-scavenging assays. Plants are a major source of phenolic compounds such as cinnamic, p-coumaric, caffeic, ferulic, chlorogenic, protocatechuic, and gallic acids [10]. The total phenolic content assay commonly uses the gallic acid as a standard. The FRAP assay is done to determine the antioxidant activity of a compound by measuring its redox property to reduce the ferric ion by single electron donation, while the DPPH radical-scavenging assay measures the ability of the compound to donate hydrogen to DPPH radical. There is a strong positive correlation between the FRAP and DPPH assays [30, 31] and between both assays and the total phenolic content [31].

Table 3 summarizes screening studies of the antioxidant properties of *P. speciosa*. The antioxidant capacity was relatively very high in the pods and seeds mixture where the methanolic extract had larger capacities than the aqueous extract for all the three assays [32]. Methanolic extract contains hydrophilic and intermediate hydrophilic compounds, whereas the aqueous extract contains hydrophilic constituents only. This difference explains the greater capacity of the methanolic extract. Antioxidant activity was also present in the seeds and leaves of the *P. speciosa* but with lower activities when compared to the activity in the pod and seed mixtures. This suggests that the pods retain greater antioxidant content than the other parts of the plant. Flavonoids which possess antioxidant property were detected in the ethanolic extract of the seeds [10], but none was found in its methanolic extract [18]. The antioxidant content and activity of *P. speciosa* is considered high amongst edible plants [10, 32, 33] especially its total phenolic contents. There was a difference in the total phenolic and flavonoid contents from the seeds extracted in ethanol and methanol. Both compounds were found to be higher in the ethanolic extract than the methanolic extract [10, 18], which could be due to more hydrophobic compounds being retained in the ethanol compared to the methanol extract. This is further shown in two different studies [18, 34] where the total phenolic content was totally absent in the former study [18] but detected at a larger content in the latter study [34]. There are many factors that may affect the chemical content or composition in plants such as species, method of extraction, storage condition, and season and age of the plant parts at the time of harvest as well as geographical factors. 

The DPPH radical-scavenging activity of the plant was also determined based on median inhibition concentration (IC_50_). The pod ethanolic extract of the plant exhibited mean IC_50_ of 10.03 *μ*g/mL compared to those of standard antioxidants used, quercetin (0.45 *μ*g/mL), and butylated hydroxytoluene (BHT, 3.47 *μ*g/mL). The finding indicated that the extract possessed a relatively high antioxidant property when compared to other plant extracts which gave IC_50_ in the range of 0.06 to 2016.64 *μ*g/mL [35]. Another similar study showed a lower DPPH radical-scavenging mean IC_50_ of which was 0.667 *μ*g/mL in the ethanolic extract of the seeds [10], suggesting that this extract also has higher antioxidant activity in the seeds.

The antioxidant activity of the plant was also assessed using a Heinz body induction using an *in vitro* model [5]. In the study, packed red cells that were mixed with acetylphenylhydrazine (a hemolytic agent) and incubated with the extracts from *P. speciosa* seed coat and pericarp (pods) showed lower Heinz body formation than other plant extracts. This indicated that the extracts were able to inhibit oxidative destruction to the erythrocytes. The seed coat extract exhibited the highest inhibitory activity, while the pericarp extract was the third highest amongst the twenty-one plants tested. The IC_50_ for the former was 3.90 mg/mL and the latter was 46.29 mg/mL. The inhibitory activity was found to be positively correlated to the tannin concentration in the plants (*r* = 0.658, *P* < 0.01). This proved that tannin which was found in the plant extracts had a strong Heinz body inhibitory ability and antioxidant ability [5]. The findings of the study showed that *P. speciosa* has a potential to be used as an agent to reduce hemolytic jaundice.

### 4.2. Hypoglycemic Activity

Diabetes is a general term that describes a disease with an elevation of blood glucose more than normal or known as hyperglycemia, when the body is unable to metabolize glucose properly. There are two types of diabetes mellitus; insulin-dependent (IDDM, type 1) and noninsulin-dependent (NIDDM, type 2). The former type is manifested by a low insulin release due to destruction of the pancreatic cells and it can be controlled by regular administrations of insulin [36]. In type 2 NIDDM, the body fails to use insulin properly or becomes less responsive to insulin [37].

Many plants have been screened for its hypoglycemic property. Studies regarding the hypoglycemic property of *P. speciosa* had started in early 1990s [38, 39]. The plant showed good hypoglycemic activity in *in vivo* and *in vitro* experiments. Many crude *P. speciosa* extracts from the empty pods (pericarp) and seeds were tested for the activity. Research works done by Jamaluddin and Mohamed [38] and Jamaluddin et al. [40] showed that the antidiabetic activity was only observed in the chloroform extract, either in seeds or empty pods, and none in other extracts such as petroleum ether, dichloromethane, ethyl acetate, ammoniacal chloroform, and methanol. Oral administration of the chloroform extract of the empty pods and seeds significantly decreased glucose level 2 hours after ingestion and the effect lasted for at least 24 hours in alloxan-induced diabetic rats. The activity was higher in the seeds than in the empty pods [38] with a minimum effective dose of 25 mg/kg and 50 mg/kg, respectively [39, 40]. *β*-Sitosterol and stigmasterol, the two major phytosterols present in the seeds of *P. speciosa* were responsible for the hypoglycemic activity. They acted synergistically but no hypoglycemic effect was observed when they were tested individually [39], while in the empty pods, stigmast-4-en-3-one was identified and elucidated to be the active compound that produced the effect. It reduced blood glucose level by 84% at 100 mg/kg body weight compared to 111% reduction by glibenclamide (5 mg/kg body weight) [40]. However, the possible hypoglycemic mechanism of the pure compounds whether they had any effect on insulin release or glucose absorption from the gut was not elucidated in these studies.

Other than alloxan-induced diabetic rat model, the hypoglycemic property of the plant was also tested in *in vitro* experiments by measuring the activities of *α*-amylase and *α*-glucosidase. *α*-Amylase is an enzyme that is involved in carbohydrates breakdown to produce simpler saccharides, whereas *α*-glucosidase is the enzyme involved in the carbohydrates intestinal absorption [41]. Therefore, inhibition of both enzymes would be beneficial in NIDDM treatment due to the delayed digestion and uptake of glucose from the intestinal tract. Decreased postprandial hyperglycemia is one of therapeutic approaches in the management of diabetes. Strong inhibitor of both enzymes like Acarbose [42] produces common side effects such as abdominal distention and flatulence, due to its strong inhibition on pancreatic *α*-amylase [43], thus results in colonic bacterial fermentation of undigested carbohydrates [44]. Therefore, a drug with a weak *α*-amylase inhibition but a good inhibitory property against *α*-glucosidase would be a good alternative.

The hexane and dichloromethane extracts of *P. speciosa* seeds showed no inhibitory effect on *α*-amylase activity [45]. However, the aqueous extract of the plant demonstrated a good *α*-glucosidase inhibitory activity with the empty pods having almost 15 times higher activity than the seeds [46, 47]. The *α*-glucosidase inhibitory activity was also observed in the petroleum ether, dichloromethane, and ethanolic extracts. Similar to the aqueous extract, the empty pods of these extracts possessed better activity than the seeds about twofold [48]. However, the aqueous extract of the seeds was able to increase insulin release [49]. 

*P. speciosa* has the potential to be developed as an oral hypoglycemic agent. Nevertheless, it was not shown to have any effect on blood glucose level in normal healthy experimental animals [38, 40]. Several possible mechanistic approaches need to be carried out before its possible use in humans.

### 4.3. Antitumor and Antimutagenicity

Cancer is one of the leading causes of death worldwide. Much attention has been paid to explore any potential antitumor agents in edible plants, for future use in humans. Amongst many medicinal plants screened, the methanolic extract of *P. speciosa* seeds demonstrated a moderate antimutagenic activity in the Ames test [34], while in inhibition assay of Epstein-Barr virus (EBV), the antitumor promoting activity of the seeds was considered as weakly active [50]. Nevertheless, it had been reported that consumption of the raw seeds reduced the incidence of esophageal cancer in Southern Thailand [51]. 

The methanolic extract of the seed coats exhibited selective cytotoxicity on breast cancer (MCG-7 and T47D), colon cancer (HCT-116), and hepatocarcinoma (HepG2) cells [52]. The methanolic extract of the seeds on the other hand did not show significant cytotoxic effect on any cancer cell lines. However, the ethyl acetate subextract of the methanolic extract showed selective cytotoxicity on hormone sensitive breast cancer cells, MCF-7 [53]. These findings are not conclusive yet due to the nature of the studies that used crude extract rather than pure compounds.

Development of a tumor is always associated with host immune response. Increased immune system enhances the ability of the host to resist tumor development as well as infectious diseases. Compounds with the ability to increase mitogenesis of lymphocytes may have a potential to be used as antitumor agents [54]. Lectin isolated from the *P. speciosa* seeds had shown mitogenic effect on human lymphocytes and rat thymocytes. The lectin stimulated incorporation of [^3^H]-thymidine into cell DNA [55, 56]. Its activity increased with the increasing dose, before declining to an optimum point. The effect was comparable to other known T-cell mitogens such as concanavalin A, pokeweed mitogen, and phytohemagglutinin [55]. Lectins with mitogenic activity usually exert antiproliferative, immunomodulatory, and antitumor properties [54]. Similar to lectins from other sources, the *P. speciosa* lectin also had a strong hemagglutinating activity for rabbit, goat, rat, and human erythrocytes [47, 56, 57]. These findings indicate that lectin from the seeds may increase DNA synthesis and, therefore, may enhance immune response against infections and tumors. The effect of natural products on tumor cell viability was found to be negatively associated with their mitogenic activity [58]. 

Angiogenesis is a critical process which is involved in many physiological and pathological conditions such as metastasis of solid tumors. The methanolic extract of the fresh pods of the *P. speciosa* demonstrated antiangiogenic activity. *In vitro*, the extract inhibited microvessel outgrowth in rat aortae more than 50%, which was not observed in the water and hexane extracts. It also inhibited the ability of human umbilical vein endothelial cells (HUVEC) to form capillary-like structures in matrigel matrix [53]. Both extracts of hexane and methanol of the seed coats showed antiangiogenic activity in rat aortic rings with vessels outgrowth inhibition of 74% and 82%, respectively [52]. The effect might be due to the formation of many vacuoles in the endothelial cells observed in light microscopy [53]. The presence of the vacuoles indicates cellular starvation due to nutritional deprivation which is an essential characteristic to maintain the viability of the cells [59]. This property is beneficial in the treatment of cancer due to its ability to prevent neovascularization of the tumors.

Thiazolidine-4-carboxylic acid, a thioproline, might be responsible for the antitumor effect of the seeds. It was shown to possess antiproliferative effects against cancer cells [22]. It was found in cooked seeds of *P. speciosa* but was undetectable in uncooked seeds [21]. The compound is an effective nitrite-trapping agent which can inhibit the endogenous formation of carcinogenic N-nitroso compounds [60]. Its derivatives were previously designed and synthesized as novel influenza neuraminidase inhibitors [61]. 

### 4.4. Antimicrobial Activity

The seeds of *P. speciosa* have been used by the Orang Asli in West Malaysia to treat kidney disorder which is believed to be urinary tract infection [1]. Studies regarding the antimicrobial property of *P. speciosa* are still lacking where, so far, only the seeds of the plant have been screened for its antimicrobial activity. The extracts of the seeds in petroleum ether, chloroform, and methanol demonstrated antibacterial activity against *Helicobacter pylori* but none was found in the water extract. The activity was the highest in the chloroform extract followed by methanol and petroleum ether. Comparatively, the chloroform extract showed a moderate inhibition zone diameter to mg extract ratio (25.0), while the ratio of other plant extracts was in the range of 1.5 to 117.5 [62]. A previous study also showed the ability of the seed extract in methanol to inhibit *H. pylori* growth, while the ethyl acetate extract was effective against *Escherichia coli*. These extracts, however, had no inhibitory effect on *Salmonella typhimurium*, *Salmonella typhi,* and *Shigella sonnei* growth [63]. An aqueous suspension of the seeds displayed an ability to inhibit the growth of *Aeromonas hydrophila*, *Staphylococcus aureus*,* Streptococcus agalactiae*,* Streptococcus anginosus,* and *Vibrio parahaemolyticus*. However, the suspension was ineffective against *Citrobacter freundii*, *Edwardsiella tarda*, *Escherichia coli*,* Vibrio alginolyticus*, and *Vibrio vulnificus.* These bacteria were isolated from moribund fishes and shrimps [64]. 

Collectively, it may be assumed that the seeds of *P. speciosa* are more effective against Gram-negative bacteria. However, the spectrum of the activity depends on the type of the extract. The antibacterial property is due to the seed content of hexathionine and trithiolane, two cyclic polysulfide compounds [19]. It was also screened for antiplasmodial activity against *Plasmodium falciparum,* but no activity was found [65].

### 4.5. Effects on Cardiovascular System

Decoction of the roots of *P. speciosa* has been used in folk medicine in Malaysia to treat hypertension [9, 66]. To date, however, no scientific data regarding the plant effects on hypertension is available. Hypertension increases the risk of atherosclerosis, an artery clogging process which leads to heart attacks and strokes. Angiogenesis plays an important role in atherosclerosis. As mentioned earlier, the methanolic extract of the empty pods also possessed antiangiogenic property. It is suggestive that the plant extract may inhibit or reduce the development of atherosclerosis, thus needs to be explored further. Vascular endothelial growth factor (VEGF) is a factor that is involved in pathological angiogenesis or hypervascularization [67] which also plays a crucial role in atherosclerotic lesions [68]. The methanolic extract was shown to inhibit the expression of VEGF and neovascularization in rat aortic rings [53]. 

Its possible fibrinolytic activity was screened among other Thai indigenous plants using an *in vitro* experiment by measuring the clear zone area of fibrinogen and thrombin mixture. Relatively, *P. speciosa* had the lowest fibrinolytic activity which was only 1.5 mm^2^ (the plant with the highest activity was 50.2 mm^2^) [69]. Thus, it can be considered that the plant has no significant fibrinolytic activity.

## 5. Pharmacokinetics and Toxicity

To date there is no single study conducted on the pharmacokinetics of *P. speciosa*. This could be due to research being carried out on this plant so far only used its crude extracts rather than the pure compounds. A few studies conducted had detected the active compounds responsible for its hypoglycemic effects (*β*-sitosterol, stigmasterol, and stigmast-4-en-3-one), antibacterial activity (hexathionine and trithiolane), and antitumor (lectin and thiazolidine-4-carboxylic acid) [19, 39, 40, 56], but to date no study had used the pure compounds for the respective effects. Other studies performed were still at the stage of activity screening. Pharmacokinetic data of *P. speciosa* is important and vital in order to better understand its pharmacodynamic effects. The extent of its absorption would affect the amount of dosage needs to be administered. Its metabolism pathway should also be studied to determine whether the produced metabolites are toxic or otherwise. Its excretion is believed to be through the kidneys due to the odorized urine after its consumption. Other possible routes are not known. 

Its toxicity study is also lacking. No *in vivo* toxicity study has been carried out. Only Aisha et al. [53] had performed a cytotoxicity study of the plant using HUVEC. In their study, the methanolic extract of the fresh pods (100 *μ*g/mL) did not show any significant cytotoxic effect on the cell lines. Information gathered from the locals, consumption of the seeds up to 30 seeds (two long pods) in a serving almost everyday does not cause any adverse effect. 

## 6. Conclusion

*Parkia speciosa* Hassk. which is rich in antioxidant content especially total phenolic has many potentials to be developed as a phytomedicine. The properties are attributable to the presence of *β*-sitosterol, stigmasterol, stigmastenone, thiazolidine-4-carboxylic acid, hexathionine, and trithiolane in the plant. Traditionally, it is used to treat hypertension, diabetes, and headache, but with no scientific evidence so far. Many scientific studies have been performed on its hypoglycemic, antitumor, antimicrobial, and antiangiogenic properties. This still warrants further studies to explore the potential properties of the plant including its antihypertensive, analgesic, or anti-inflammatory (due to its lupeol and flavonoid contents) properties. Further toxicity studies and research in humans should also be conducted.

## Figures and Tables

**Table 1:** Nutritional value of *Parkia speciosa* Hassk. seeds.

Component	Composition (per 100 g edible portion)
Ash (g)	1.2–4.6
Protein (g)	6.0–27.5
Fat (g)	1.6–13.3
Carbohydrate (g)	13.2–52.9
Crude fiber (g)	1.7–2.0
Energy (kcal)	91.0–441.5
Calcium (mg)	108.0–265.1
Iron (mg)	2.2–2.7
Phosphorus (mg)	115.0
Potassium (mg)	341.0
Magnesium (mg)	29.0
Manganese (ppm)	42.0
Copper (ppm)	36.7
Zinc (ppm)	8.2
Vitamin C (mg)	19.3
*α*-Tocopherol (mg)	4.15
Thiamin (mg)	0.28

References: [10–12, 70].

**Table 2:** Phytochemical substances in *P. speciosa*.

Parts	Alkaloid	Saponin	Terpenoids	Phenolic	Flavonoid	Tannin
Seeds	+	−	+	+	+	−
Barks	+	−	−	+	−	ND
Leaves	−	−	+	+	+	ND
Seed coats	+	+	−	−	+	+
Pods	ND	ND	ND	+	ND	+

Abbreviations: +: present; −: absent; ND: not determined. References: [5, 10, 32, 71].

**Table 3:** Antioxidant activity in various *P. speciosa* extracts.

Plant part	Extract	Total phenolic content (mg GAE/g)^a^	DPPH assay (*μ*mol Trolox/g)^a^	FRAP assay (*μ*mol Trolox/g)^a^	Total flavonoids (mg RE/g)^a^	Tannin (mg/g)^a^	Reference
Pod and seed	Aqueous	1557.6^b,c^	7418.3^b,d^	1617.3^b,d^	—	—	Ayub Ali et al. [32]
Pod and seed	Methanol	2464.3^b,c^	5936.9^b,d^	1898.0^b,d^	—	—	Ayub Ali et al. [32]
Pod	Ethanol	—	—	—	—	250	Tunsaringkarn et al. [5]
Seed	Ethanol	51.9^a^	—	—	20.3^a^	—	Maisuthisakul et al. [10]
Seed	Methanol	—	—	—	0	—	Miean and Mohamed [18]
Seed	Methanol	120^b,c^	40^b,e^	—	—	—	Tangkanakul et al. [34]
Seed	Aqueous	6.5	67.62	44.67	—	—	Reihani and Azhar [30]
Seed coat	Ethanol	—	—	—	—	350	Tunsaringkarn et al. [5]
Leaf	Ethanol	44.7	89.26^f^	—	—	—	Tangkanakul et al. [4]
Leaf	Aqueous	22.7	57.4^f^	—	—	—	Tangkanakul et al. [4]
Leaf	Aqueous	32.73	22.7	49.9	—	—	Wong et al. [33]

^a^Dry weight basis, ^b^fresh weight basis, ^c^(mg GAE/100 g), ^d^(mg Trolox/100 g), ^e^(mg vitamin C equivalent/g), and ^f^(mg BHA equivalent/g).
